# A UK key opinion leader perspective: Navigating the immunological and logistical transformation brought by stem cell‐derived islets for the treatment of type 1 diabetes

**DOI:** 10.1111/dme.70230

**Published:** 2026-01-20

**Authors:** Thomas Strakosch, Shareen Forbes

**Affiliations:** ^1^ Cell and Gene Therapy Catapult (CGTC) London UK; ^2^ BHF Centre for Cardiovascular Science, Institute for Neuroscience and Cardiovascular Research, Queen's Medical Research Institute University of Edinburgh Edinburgh UK

**Keywords:** calcineurin inhibitors, immunosuppression, islet and kidney transplantation, islet transplantation, stem cell derived islets, type 1 diabetes

## Abstract

**Aims:**

To explore UK key‐opinion leader perspectives on the future role of stem cell‐derived islets (sc‐islets) in islet transplantation for people with type 1 diabetes (T1D).

**Methods:**

Four UK‐based key‐opinion leaders evaluated current limitations of donor islet transplantation and reviewed emerging evidence, clinical pathways and logistical considerations for sc‐islet transplantation, including alternative delivery sites and implications for kidney transplantation strategies.

**Results:**

Conventional islet transplantation is constrained by donor scarcity, variable graft quality and lifelong immunosuppression, with associated risks of infection, malignancy and calcineurin inhibitor (CNI) nephrotoxicity. Stem cell‐derived islets, generated from human embryonic and induced pluripotent stem cells, provide a scalable and standardised alternative. Early investigational products, including Zimislecel (VX‐880), demonstrate potential for insulin independence and may offer an alternative to simultaneous pancreas–kidney (SPK) transplantation. Strategies to reduce or eliminate systemic immunosuppression particularly CNI immunosuppression through local immunomodulation, gene editing and encapsulation technologies may further broaden access. Ethics, infrastructural and economic considerations remain central to equitable implementation.

**Conclusion:**

Stem cell–derived islets may redefine islet transplantation for T1D by enabling more scalable, less invasive and sustainable therapeutic pathways while maintaining access to technological diabetes management options.

**What's new?**

- This UK key opinion leader perspective highlights how sc‐islets could shift islet transplantation from a donor‐limited therapy to a scalable, manufactured treatment platform.
- The work outlines how sc‐islets may fundamentally alter current kidney–pancreas transplant hierarchies, potentially challenging the future role of SPK transplantation.
- Calcineurin inhibitor nephrotoxicity is identified as a central barrier to wider adoption of beta cell replacement therapy.
- The elimination of systemic immunosuppression using gene editing, encapsulation and local immunomodulation is highlighted as a key enabler of broader, equitable access.

## 1 INTRODUCTION

Islet transplantation, whereby islets are isolated from deceased donor pancreases and infused into the liver in order to stabilise blood glucose control, is a procedure that has been recommended by the National Institute for Health and Care Excellence (NICE) since 2008 for a subset of adults with type 1 diabetes (T1D) with problematic glycaemic control.^1^, ^2^ This therapeutic intervention requires immunosuppression which is associated with an increased risk of infections and cancers. Other co‐morbidities including hypertension^3^ and dyslipidaemia^4^ may also occur but these tend to be associated with high doses of sirolimus.^5^ As such, this intervention is therefore only considered as a stand‐alone procedure where diabetes management has been optimised.^6^ The intervention is particularly pertinent in the context of people with T1D and renal failure: the majority of people now listed for an islet transplantation in the UK are those requiring both islets and a kidney transplant (KT).^7^

The future of islet transplantation with pluripotent stem cell‐derived islets (sc‐islets) in people living with T1D brings with it both opportunities and challenges for recipients and the wider medical establishment. Four UK‐based key‐opinion‐leaders (KOL), including two clinical diabetologists with expertise in islet and combined islet and kidney transplantation, a surgeon with expertise in islet, pancreas and kidney transplantation and a scientist with expertise in regenerative medicine for diabetes and transplant immunology, discussed the future of islet transplantation with sc‐islets in people living with T1D.

Detailed semi‐structured interviews were conducted from January 2025 to February 2025 by an independent party utilising a pre‐prepared discussion guide. Fourteen KOLs were considered prior to the request for interview. Of those contacted, half (four of eight) responded with agreement to participate. The opinions were analysed and information was synthesised into a report and supporting literature added. The collective opinions are represented below.

## 2 SHIFTING THE PARADIGM OF BETA CELL REPLACEMENT THERAPY

The management of T1D has benefited substantially from technological advancements, yet the definitive clinical aspiration remains the restoration of physiological, endogenous insulin production, effectively delivering a cure. For patients facing End‐Stage Renal Disease secondary to diabetic nephropathy, the established hierarchy of care places simultaneous pancreas‐kidney (SPK) transplantation as the first‐choice intervention, offering the greatest potential for reversing diabetes complications and achieving the best long‐term insulin independence rates. However, SPK eligibility is highly restrictive, dictated by stringent criteria concerning the recipient's BMI and age (typically <30 kg/m^2^ and <60 years respectively) as well as freedom from significant cardiopulmonary co‐morbidities.^8^, ^9^, ^10^

For patients deemed unsuitable for SPK, or those whose deteriorating renal function declines to the point a combined transplant procedure becomes mandatory (e.g. eGFR <20 mL/min/1.73m^2^),^11^ the alternatives include simultaneous islet and kidney (SIK) or islets after kidney (IAK) transplantation^12^ which impacts glycaemic control more favourably than kidney transplant alone (KTA).^13^ In the context of normal renal function, as of September 2025, Islet transplant alone (ITA) is reserved for a select subset of patients who have either been assessed by a diabetologist to have disabling hypoglycaemia (defined by NICE to be ‘repeated and unpredictable occurrence of hypoglycaemia that results in persistent anxiety about recurrence and is associated with a significant adverse effect on quality of life’), or to have an HbA1c >58 mmol/mol despite optimised conventional therapy.^14^, ^15^, ^16^, ^17^ A critical observation regarding these existing pathways is that the current treatment hierarchy is dictated not primarily by achieving optimal long‐term metabolic stability but rather by logistical constraints, namely, the profound scarcity and variable quality of deceased donor pancreases.^7^, ^18^, ^19^ The allocation system further reflects the perceived urgency of metabolic control, evidenced by the shorter deceased donor waiting list for SIK (1.7 years) compared to KTA (2.5 years), which may influence the clinical decision to prioritise combined organ approaches.^7^ The situation is compounded by the need for multiple islet transplants in one individual due to a combination of donor and recipient factors, including poor numbers of islets retrieved as well as alloimmune, autoimmune and inflammatory mediated islet apoptosis following transplantation.^20^ Over time there is attrition in islet graft function. Multiple transplants are therefore required sequentially: initially patients typically receive two islet transplants within a 3‐month period^21^ but may then go on to receive top‐up transplants years later.^22^

This reliance on finite donor resources has created an inflexible pathway, underscoring the urgent need for scalable alternatives.

## 3 THE TRANSFORMATIVE PROMISE OF SCALABLE CELL THERAPY WITH HUMAN SC‐DERIVED ISLETS

The advent of human embryonic stem cell‐derived (hESC) islets^23^ and more recently human induced pluripotent stem cell‐derived (hiPSC) islets^24^, ^25^, ^26^ offers a pathway to fundamentally transform T1D treatment from organ‐based surgery to scalable, manufactured cell therapy. Investigational products, such as Zimislecel (VX‐880) from Vertex Pharmaceuticals, consisting of allogeneic hESC‐derived fully differentiated pancreatic islets, have demonstrated in Phase 1/2 trials the capacity to restore physiologic islet function in patients with severe hypoglycaemia, with 10 of 12 participants (83%) achieving insulin independence.^23^ This development addresses the most significant constraint in current beta cell replacement: the scarcity, unpredictability and variable quality of donor pancreases.^18^

Implementation of a reproducible manufacturing paradigm^27^ enhances quality control and may improve graft durability and functional outcomes, potentially minimising the number of islet infusions needed to achieve insulin independence in conventional ITA.^21^ Furthermore, the transition away from dependence on variable‐quality cadaveric organs allows for greater timeliness and precision in dosing, ensuring that an optimal initial graft is delivered without the unpredictability associated with donor availability.^28^, ^29^

**FIGURE 1:**
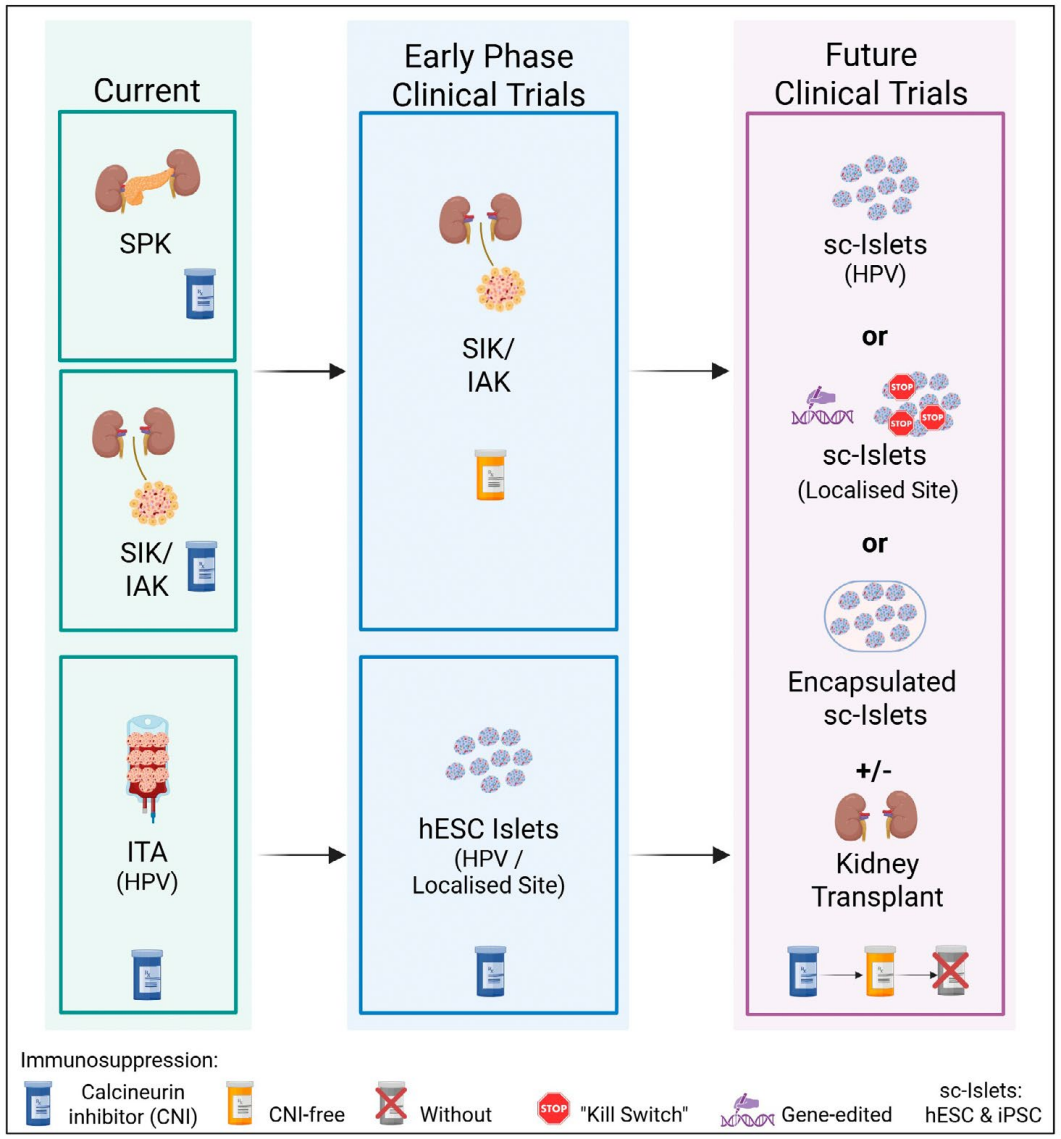
Current and future transplant pathways for people with T1D. Currently, in select adults with renal failure and type 1 diabetes (T1D), simulataneous pancreas‐kidney (SPK) transplant offered to adults that meet age, BMI and fitness criteria; otherwise simultaneous islet and kidney (SIK)/islets after kidney (IAK) transplant offered. Islet transplant alone (ITA) is offered to adults with severe hypo‐glycaemia and glycaemic instability where treatment is optimised and CNI‐associated immunosuppression is given. Early phase clinical trials have shown success of islet transplantation into the liver in people with a kidney transplant under calcineurin inhibitor (CNI)‐free immunosuppression^42^ and of human embryonic stem cell (hESC) islet transplantation into the liver with CNI immunosuppression administered.^17^ In the next stage of clinical trials, sc‐islet transplantation with or without kidney transplantation is done under CNI immunosuppression with evolution to CNI‐free immunosuppression. In the context of sc‐islet transplantation alone, clinical trials are anticipated to be done with CNI‐free immunosuppression with evolution to immunosuppression‐free protocols either with gene‐edited hypoimmune islets^19^ or encapsulated islets^48^ with potentially local immunomodulation.^49^ CNI, calcineurin inhibitor; HPV, hepatic portal vein; hESC, human embryonic stem cell; IAK, islets after kidney; ITA, islet transplant alone; SC, stem cell; SIK, simultaneous islet and kidney; SPK, simultaneous pancreas–kidney; T1D, type 1 diabetes.

Looking ahead, advances in gene editing and encapsulation technologies could eliminate the need for immunosuppression, enabling this therapy to be extended to children, as discussed below. Figure 1, outlines a potential road map to the clinic.

## 4 LOGISTICAL SHIFTS AND PROCEDURAL INNOVATION

Current islet transplantation procedures are heavily constrained by infrastructure, including the limited availability of interventional radiologists who perform the necessary portal vein infusion and the unpredictable nature of deceased donor islet isolation, which limits scheduling efficiency. The availability of an ‘off‐the‐shelf’ stem cell supply, offering predictable, on‐demand availability, resolves this logistical challenge allowing for advance planning, targeted resource allocation and workforce training, effectively expanding the capacity for beta cell transplantation.^30^

Moreover, the regenerative medicine landscape is fostering innovation in delivery methods, moving away from the complex portal vein infusion technique to alternative sites for cell implantation, such as the greater omentum,^31^ muscle^25^ or devices like the Cell Pouch,^32^ which require initial surgical implantation followed by subsequent islet infusion into a vascularised chamber. These alternative delivery systems could in theory simplify and reduce the risk profile of the procedure, expand the options as to where the treatment can be carried out, and, due to the localised position of the graft, ensure that graft surveillance could be more effectively carried out. However, persistent challenges, including limited reproducibility and poor durability of transplanted islet function at extra‐hepatic sites, have hindered the successful translation of preclinical work into efficacious human therapies.^33^

A final, crucial implication of this scalability involves the future burden of chronic care. Although high‐quality stem cell grafts are expected to decrease the need in the short term for urgent ‘fast‐tracked’ top‐up infusions, the overall demand for repeat transplants is projected to rise. This is because wider eligibility will lead to more patients undergoing the procedure and islet function, even with human islet donor grafts, typically wanes after >5 years^22^, ^34^ although large variability exists.^35^ The long‐term resource burden will thus shift from managing initial scarcity to planning infrastructure capable of supporting chronic maintenance therapy through planned top‐ups under existing maintenance immunosuppression.

## 5 SC‐ISLETS IN THE CONTEXT OF KIDNEY TRANSPLANTATION

A high‐quality, unlimited supply of sc‐islets is anticipated to fundamentally alter the hierarchy of treatment. If sc‐islets can achieve metabolic stability and insulin independence comparable to pancreas grafts, while avoiding the significant surgical risks and substantial early graft failure rates associated with whole‐organ pancreas transplantation,^36^, ^37^ the clinical utility of SPK could be fundamentally questioned. This suggests that, providing insulin independence can be achieved reliably with transplantation of sc‐islets, SPK may eventually be phased out and replaced by the far less invasive combined sc‐islet and KT approach. This strategy is particularly compelling when paired with living donor kidney (LDK) transplantation, which has excellent long‐term outcomes and short waiting times (typically 4 months).^38^ However, if insulin independence rates are less than with an SPK, then SPK may be the preferred option; nevertheless, there is a complex interplay between success, availability and wait time.

However, the sc‐islet + KT strategy differs critically from conventional SIK, where both organs share the same donor, offering a potential, though often minor in practise, immunological advantage.^39^

**FIGURE 2:**
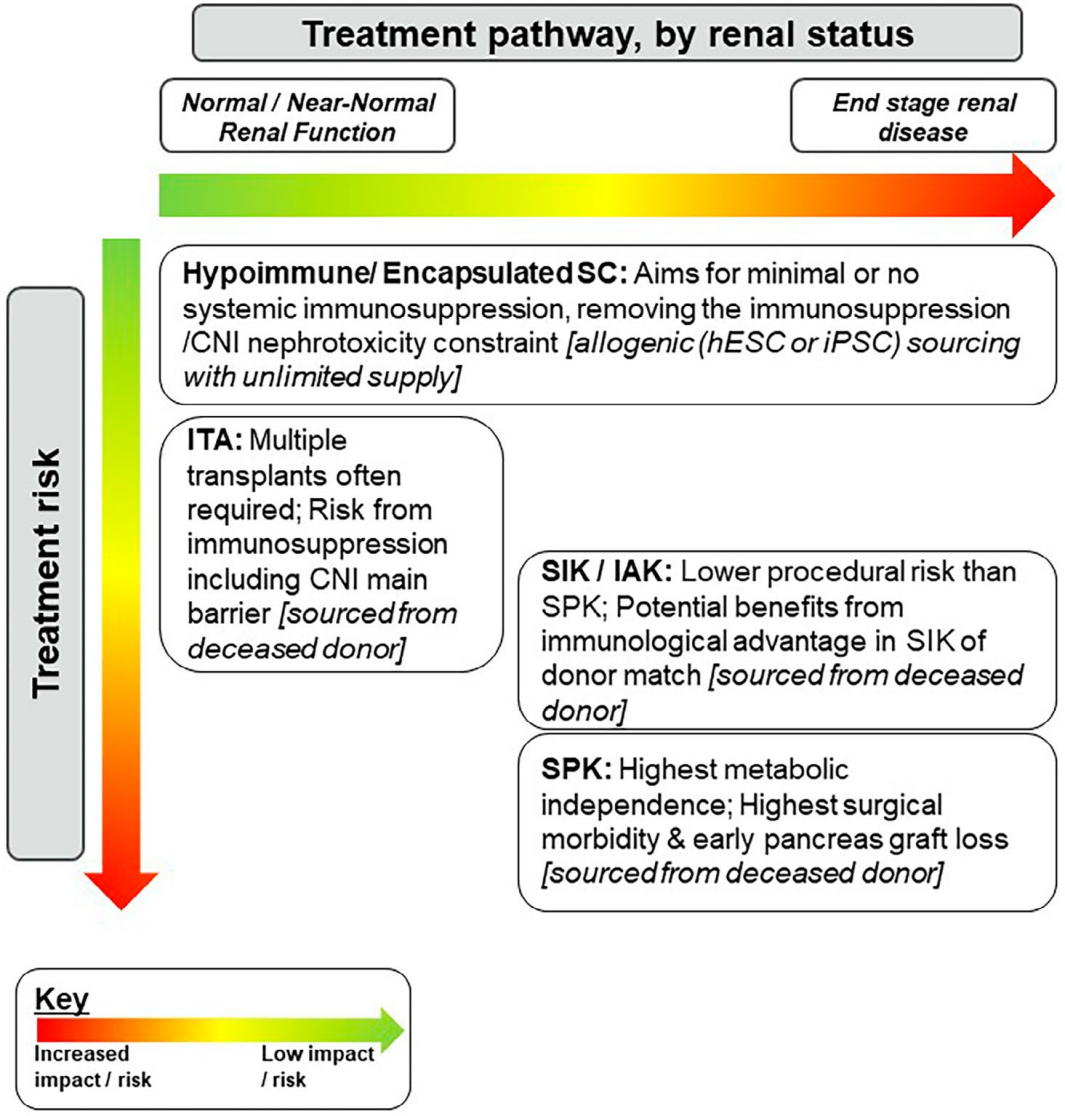
Treatment pathway by renal status for people with T1D. currently, simultaneous pancreas‐kidney (SPK), simultaneous islet and kidney (SIK), and islets after kidney (IAK) are offered to adults with renal failure and type 1 diabetes (T1D). These transplant options are associated with higher morbidity than islet transplant alone (ITA), which is offered to individuals without significant renal impairment. Emerging approaches using hypoimmune and/or encapsulated stem cell‐derived islets may expand eligibility to people with T1D regardless of renal status and, with the potential for an unlimited cell supply and reduced immunosuppression, may offer a more favourable benefit–risk profile. CNI, calcineurin inhibitor; hESC, human embryonic stem cell; IAK, islets after kidney; iPSC, induced pluripotent stem cell; ITA, islet transplant alone; SIK, simultaneous islet and kidney; SPK, simultaneous pancreas–kidney; stem cell (includes iPSC or hESC).

In the sc‐islet + KT scenario, the islet and kidney components are non‐matched, introducing unique immunological complexities. To mitigate risks, a sequenced approach could be considered: the KT could be performed first to ensure organ acceptance and stabilisation of the immunosuppressive regimen, followed by the sc‐islet infusion shortly after under the established induction and immunosuppression window. Figure 2 summarises the transplant pathway by renal status for people with T1D.

## 6 THE IMMUNOLOGICAL COST OF BETA CELL RESTORATION

The primary clinical barrier to expanding islet transplantation remains the need for systemic immunosuppression.^20^ Currently in islet transplantation, induction therapy is required to prevent graft rejection, and these are tailored to regional practises. In the United Kingdom, Alemtuzumab, a monoclonal antibody targeting CD52 on mature lymphocytes, is frequently used for ITA, whereas in the United States, anti‐thymocyte globulin (ATG) is commonly used in stem cell trials.^40^, ^41^ This T‐cell depletion therapy is associated with transient but marked leucopenia and lymphopenia. For patients with multiple co‐morbidities, Basiliximab may be more appropriate, but by itself does not reliably induce tolerance.^42^, ^43^ Basiliximab targets the CD25 alpha subunit of the IL‐2 receptor on activated T‐cells, inhibiting their proliferation and avoiding profound lymphopaenia, which is associated with an increased risk of infection. Lifelong maintenance immunosuppression is required to prevent graft rejection and typically consists of mycophenolate mofetil (MMF), which inhibits inosine monophosphate dehydrogenase, an enzyme essential for de novo guanosine nucleotide synthesis on which proliferating T and B lymphocytes heavily depend, together with the calcineurin inhibitor (CNI) tacrolimus. Non‐compliance with this regimen invariably results in graft loss. Although side effects are often managed, such as opting for the enteric coated mycophenolic acid over MMF to alleviate gastrointestinal issues, the persistent risk of infections, malignancies and post‐transplant lymphoproliferative disorder remain significant barriers to widespread therapy adoption.^29^ CNIs pose particular challenges and may induce insulin resistance, with potential impact on graft function,^44^ exert direct toxic effects on beta cell function^45^ and suppress regulatory T cells, increasing the risk of alloimmune activation and chronic rejection^46^ as well as nephrotoxicity, discussed further below. As such, islet transplantation is currently limited to adults with T1D, highlighting the need to eliminate immunosuppression to make it accessible to a broader population. Data from the Collaborative Islet Transplant Registry (CITR) indicate that the cancer‐related risk associated with immunosuppression is low.^34^ Importantly, the perceived risk of immunosuppression, rather than the actual risk, may represent a greater barrier to broader adoption, and this is likely to vary depending on the baseline risk of the population.

## 7 THE CNI NEPHROTOXICITY DILEMMA

While the long‐term metabolic benefits of restored endogenous insulin production can be protective, particularly for micro‐vascular health,^47^ the nephrotoxic potential of the CNI regimen must be weighed against the patient's existing renal status, necessitating lifelong kidney function monitoring.^48^ The risk profile associated with these agents makes ITA inappropriate for patients with significant and progressive renal dysfunction unless concurrent KT is performed, as the immunosuppression may accelerate underlying renal decline.^48^ The potential for sc‐islets to be offered as an adjunctive therapy to LDK recipients necessitates a careful assessment of the risk–benefit balance. The nephrotoxicity associated with CNIs requires the translation of CNI‐sparing protocols into standard clinical practise and is now an area of intensive research.^49^

## 8 ACCESS, ELIGIBILITY AND MONITORING

The potential for an unlimited sc‐islet supply introduces complex ethics and equity considerations regarding kidney allocation.^50^ In the United Kingdom, current clinical practise allows for earlier access to kidneys in SIK recipients (eGFR <20 mL/min) compared to patients receiving KTA. If the predictable availability of sc‐islets were leveraged to justify moving kidneys further up the treatment pathway solely to facilitate cell therapy,^13^ it would raise significant equity‐of‐access concerns within the transplant community, potentially distorting allocation priorities. Furthermore, monitoring transplant success in renal cohorts is challenging. C‐peptide concentrations, the accepted metric for assessing beta cell function, can be falsely elevated in patients with renal failure, as C‐peptide is eliminated via the kidneys.^51^ This complicates the interpretation of the C‐peptide contribution from the grafted islets, particularly as renal function recovers post‐KT.

Composite metrics, such as the BETA‐2 score which includes C‐peptide, glucose, HbA1c and insulin requirements,^52^ and the Igls score which also incorporates severe hypoglycaemic events,^53^ are essential to use across the disciplines of pancreas, islet and stem cell transplants in order to provide objective measures of graft function that can be compared.

## 9 THE PATH TO IMMUNOLOGICAL TOLERANCE

The ultimate goal of regenerative medicine in T1D is the elimination of systemic immunosuppression. Advances in gene editing technologies, particularly CRISPR/Cas9, now enable the precise modification of human pluripotent stem cell‐derived beta cells to create hypoimmunogenic islets. These cells can be engineered to express human leukocyte antigen (HLA)‐negative profiles and overexpress immunoregulatory factors such as CD47 and PD‐L1, thereby evading T‐cell and natural killer (NK) cell‐mediated responses. In a single case study hypoimmune islets achieved insulin independence in a participant for 6 months, without systemic immunosuppression.^25^ Although promising, such studies have not as yet entered clinical trials and long‐term insulin independence rates are not known. Nevertheless, this research, alongside efforts to induce immunological tolerance through donor haematopoietic stem cell infusion in kidney recipients,^54^, ^55^ represents a pathway toward removing the CNI‐related renal risk entirely. However, ongoing safety concerns related to immune escape, viral infection risk and tumorigenicity (‘rogue stem cells’) must be robustly addressed before widespread clinical use, potentially requiring the integration of a cellular ‘off switch’. The higher failure rate of conventional islet transplants compared to pancreas grafts highlights a critical need for new monitoring tools. If ‘naked’ hypoimmune sc‐islets are used, the absence of this immunological sentinel will necessitate the development of highly sensitive, non‐invasive methods to detect subclinical islet graft rejection early. An area of active research are trials of islets within immunoprotected devices. In the recent VX‐264 study, individuals with T1D received differentiated pancreatic islets encapsulated in a proprietary device without immunosuppression.^56^ Although the therapy was well tolerated, C‐peptide levels at Day 90 were not clinically meaningful, and the trial was stopped early, underscoring both the promise of device‐based delivery and the remaining challenge of achieving functional insulin secretion in the absence of immunosuppression. A further approach is of local immunomodulation with immune cells or immunomodulatory cytokines targeted to the transplant site which could diminish the doses of immunosuppression used.^57^

Table 1 summarises current and future immunosuppression strategies.

**TABLE 1:** Immunosuppression risks and mitigations.

Agent/strategy	Specific risk/Adverse effect	Translational mitigation strategy	Strategies for sc‐Islets
Alemtuzumab/ATG	T‐cell deficiency; Infection risk PTLD and malignancy risk	Use of Basiliximab in co‐morbid patients (T‐cell activation inhibitor)	Standard depletion for ‘naked cell’ therapy (e.g., Zimislecel)
Tacrolimus (CNI)	Nephrotoxicity (accelerated renal decline) Down regulates regulatory T cells Infection risk PTLD and malignancy risk	Renal‐sparing protocols (e.g., Sirolimus‐based) Tolerance inducing agents (e.g., tegoprubart)	Primary barrier to use in patients with nephropathy
MMF/Myfortic	Gastrointestinal issues Infection risk PTLD and malignancy risk	Change formulation to mycophenolic acid (Myfortic)	Contributes to overall systemic immunosuppressive burden
Gene‐Edited Islets	Immune evasion (No systemic IS required)	Potential risks: tumorigenicity, immune escape	Eliminates the renal risk balance equation entirely

Key	
	Role: induction (t‐cell depletion)
	Role: maintenance
	Role: future strategy

Abbreviations: ATG, anti‐thymocyte globulin; CNI, calcineurin inhibitor; IS, immunosuppression; MMF, mycophenolate mofetil; PTLD, post‐transplant lymphoproliferative disorder.

## 10 OTHER ASPECTS IN THE REFERRAL PATHWAY

Advances in hybrid closed loop (HCL) systems combining continuous glucose monitoring (CGM) with automated insulin delivery have markedly improved hypoglycaemia management and insulin dosing, enabling many with T1D to achieve >70% time‐in‐range (TIR) and <4% time‐below‐range (TBR), now the clinical benchmark.^58^, ^59^ For those with persistent severe hypoglycaemia, guidelines recommend structured education and, where appropriate, HCL technology before transplantation is considered. Despite technological advances, these therapies are not curative and extensive user and healthcare practitioner input is required, and they do not necessarily halt micro‐vascular disease progression, whereas transplantation can positively modify these outcomes.^60^ Clinicians must identify and refer eligible candidates early, before comorbidities preclude eligibility. Until hypoimmune stem cell transplantation is achieved, transplantation should be likely to provide a lasting metabolic benefit that clearly justifies immunosuppression over current technological alternatives.

Importantly, patient‐reported outcome measures (PROMs) and quality‐of‐life assessments specific to T1D, islet and sc‐islet transplantation need to be developed to adequately assess the impacts of these therapies and should be prospectively integrated into future trials to ensure patient‐centred outcomes inform clinical practise.^61^

## 11 CONCLUSION: DEFINING THE FUTURE LANDSCAPE AND INFRASTRUCTURAL, ECONOMIC AND ETHICS CONSIDERATIONS

The introduction of sc‐islets represents the most significant impending transformation in T1D cell therapy, promising to transition care from a model constrained by organ scarcity and high surgical risk to one defined by predictable supply and potentially lower procedural morbidity. The ability of sc‐islet transplants to attain parity with pancreas transplant efficacy, particularly in the renal cohort, could drive a fundamental shift away from SPK towards combined sc‐islet + KT. The highest priority for translational research must be directed toward fully decoupling the cell graft from toxicity of systemic immunosuppression. The development and clinical translation of hypoimmunogenic islets or functional macro‐encapsulation systems that eliminate the need for CNIs are the next steps.

Finally, expanding access will require infrastructural medical reform and sustainable economic models, potentially underpinned by partnership and contribution from the pharmaceutical industry. Full transparency regarding all risks, including infection, malignancy and the use of embryo‐derived stem cells, is essential. Furthermore, the clinical community must ensure that the availability of sc‐islets does not lead to an unethical denial of access to established HCL technologies or inadvertently exacerbate existing equity issues by inappropriately manipulating kidney allocation priorities for cell therapy facilitation. The future of T1D cell therapy lies not just in regenerative science, but in judicious, risk‐informed clinical translation that prioritises long‐term patient health and equitable access.

## 12 FUNDING INFORMATION

This study was funded by “Steve Morgan Foundation Type 1 Diabetes Grand Challenge” by Diabetes UK, Breakthrough T1D and SMF (grant number 23/0006633). Leona M. and Harry B. Helmsley Charitable Trust. Cheif Scientist Office (grant number PMAS/21/07).

## CONFLICT OF INTEREST STATEMENT

SF has collaborated with and received funding from Novo Nordisk, Copenhagen islet stem cell therapy programme and has served as an advisor to Sanofi.

## KEY OPINION LEADERS

Dr Miranda Rosenthal, King's Diabetes Centre, King's College Hospital NHS Foundation Trust, London, UK. Professor James A.M Shaw, Translational and Clinical Research Institute, Newcastle University, 4th Floor William Leech Building, Framlington Place, Newcastle upon Tyne, NE2 4HH, UK. Mr David van Dellen, Manchester Centre for Transplantation, Manchester Royal Infirmary, Manchester University NHS Foundation Trust, UK. Dr Wilson Wong, MRC Centre for Transplantation, King's College London, 5th Floor Tower Wing, Guy's Hospital, London, SE1 9RT, UK.

## Data Availability

Data sharing not applicable to this article as no datasets were generated or analysed during the current study.
